# Variable expressivity and co‐occurrence of LDLR and LDLRAP1 mutations in familial hypercholesterolemia: failure of the dominant and recessive dichotomy

**DOI:** 10.1002/mgg3.203

**Published:** 2016-02-24

**Authors:** Akl C. Fahed, Ruby Khalaf, Rony Salloum, Rabih R. Andary, Raya Safa, Inaam El‐Rassy, Elie Moubarak, Sami T. Azar, Fadi F. Bitar, Georges Nemer

**Affiliations:** ^1^ Department of Biochemistry and Molecular GeneticsAmerican University of BeirutBeirutLebanon; ^2^ Department of GeneticsHarvard Medical School and Department of Internal MedicineMassachusetts General HospitalBostonMassachusetts; ^3^ National LDL Apheresis CenterDahr El‐Bashek Governmental University HospitalRoumiehLebanon; ^4^ Department of Internal MedicineAmerican University of BeirutBeirutLebanon; ^5^ Department of Pediatrics and Adolescent MedicineAmerican University of BeirutBeirutLebanon

**Keywords:** Familial Hypercholesterolemia, *LDLRAP1*, *LDLR*, founder mutation

## Abstract

**Background:**

The familial inherited genetic disorder of lipoprotein metabolism affects more than 10 million individuals around the world. Lebanon is one of the several endemic areas for familial hypercholesterolemia (FH) with a founder mutation in the low‐density lipoprotein cholesterol receptor (*LDLR*) gene, responsible for most of the cases. We have previously shown that 16% of all familial cases with hypercholesterolemia do not show genotype segregation of *LDLR* with the underlying phenotype.

**Methods:**

We used Sanger sequencing to genotype 25 Lebanese families with severe FH for the gene encoding the LDLR‐associated protein (*LDLRAP1*), responsible for the recessive form of the disease starting with the four families that did not show any genotype‐phenotype correlation in our previous screening.

**Results:**

We showed that the previously reported p.Q136* variant is linked to the hypercholesterolemia phenotype in the four families. In addition, we showed a variable phenotype between families and between members of the same family. One family exhibits mutations in both *LDLR* and *LDLRAP1* with family members showing differential phenotypes unexplained by the underlying genotypes of the two genes.

**Conclusion:**

The p.Q136* variant in *LDLRAP1* is yet another founder mutation in Lebanon and coupled with the *LDLR* p.C681* variant explains all the genetic causes of FH in Lebanon.

## Introduction

Familial hypercholesterolemia (FH) (MIM#143890) is an inherited disorder caused by a defect that impairs the normal uptake in the liver of low‐density lipoprotein cholesterol (LDL‐C) from the blood (Khachadurian 1964; Pullinger et al. 2003; Soutar and Naoumova 2007; Kolovou et al. 2011). High LDL‐C in the blood deposits in tissues leads to a 20‐fold increase in the risk of coronary artery disease (Knowles et al. 2015). When the circulating LDL‐C levels are twice the normal values, the disease is called “heterozygous,” whereas the term “homozygous” is linked to levels that are at least fourfold higher than the normal levels (Singh and Bittner 2015). We have suggested previously that the number of mutant alleles does not always correlate with the severity of the clinical phenotype (Fahed and Nemer 2011). Based on the clinical phenotype, the prevalence of the disease has been estimated worldwide to be 1/500 for the “heterozygous form” and 1/1,000,000 for the “homozygous form” (Goldberg et al. 2011; Knowles et al. 2015; Lahtinen et al. 2015; Singh and Bittner 2015). The number of affected individuals is estimated to be more than 10 millions worldwide, with a preponderant prevalence of the “heterozygous form” in some populations like the French Canadians, Afrikaners in South Africa, Indians in South Africa, Finns, and Lebanese reaching up to 1/67 in the Danish population (Benn et al. 2012; Singh and Bittner 2015).

Mutations in three genes have been so far linked to the autosomal dominant form of the disease (ADH): the first encoding the LDL receptor (LDLR) (Brown and Goldstein 1986), the second the apolipoprotein ApoB‐100 (Soria et al. 1989), and the third the pro‐protein convertase subtilin/kexin 9 (PCSK9) (Abifadel et al. 2003). Mutations in the *LDLR* gene account for nearly 70% of all ADH cases with more than 1600 mutations reported so far (http://www.ucl.ac.uk/ldlr), and with a clinical phenotype directly linked to gene dosage, albeit not uniform even among siblings (Fahed and Nemer 2011; Singh and Bittner 2015). Those in the *APOB* and *PCSK9* account for only 2–5% of the cases in Europe, and <5% of all cases worldwide, respectively (Nissen et al. 1998; Rabes et al. 2000; Abifadel et al. 2003; Lahtinen et al. 2015; Singh and Bittner 2015). The autosomal recessive form of the disease is caused by mutations in the gene encoding the LDLR‐associated protein 1 (LDLRAP1), which is an adaptor protein that binds directly to the LDLR protein and mediates its cellular internalization via the clathrin machinery (Wilund et al. 2002; Cohen et al. 2003; Tietge et al. 2003; Pisciotta et al. 2006; Robles‐Osorio et al. 2006; Quagliarini et al. 2007; Soutar and Naoumova 2007; Soutar 2010). Similar to the case of the *LDLR*, some mutations in *LDLRAP1* are geographically linked mainly in Europe with a pronounced founder effect (Garcia et al. 2001; Wilund et al. 2002; Cohen et al. 2003; Pisciotta et al. 2006; Quagliarini et al. 2007).

The Lebanese National FH Database has recruited so far 124 patients clinically diagnosed with FH through identification of severe index cases undergoing or referred to LDL apheresis, and subsequent cascade screening of family members (Fahed and Nemer 2011; Fahed et al. 2011, 2012). The patients belong to 25 families from different parts of the country. We previously reported major findings from this database based on sequencing of *LDLR*,* PCSK9*, and *APOB* on 80 patients, and we noted that 27.5% of patients belonging to four families did not have a genetic explanation for the phenotype. In order to close this gap, we hereby report the sequencing results of all the three genes in the remaining four families in addition to those of the *LDLRAP1* genes in all patients of the database. We explain the genetic cause of FH in 100% of patients. We subsequently focus on understanding the phenotype in 44 patients belonging to four families with mutations in *LDLRAP1* gene in light of the concomitant presence of mutations for both *LDLR* and *LDLRAP1* within members of the same family.

## Methodology

### Families and subjects

The study was approved by the Institutional Review Board of the American University of Beirut. A national database of Lebanese FH patients was established at the American University of Beirut starting in 2008. Patients were recruited from the National LDL Apheresis Center, which is the only center for treatment of severe FH in Lebanon. This was subsequently followed by cascade recruitment and screening of family members. Community visits aided in recruitment of multiplex families. Blood was collected from all subjects for DNA extraction and for fasting lipid level testing. For patients on LDL apheresis, fasting lipid profile testing was collected before apheresis sessions. Clinical data were also extracted from existing medical records.

### Genetic studies

DNA extraction from blood was performed using a Qiagen kit (27220 Turnberry Lane Suite 200. Valencia, CA 91355, USA), following the manufacturer's protocol. Primers flanking all exons of the *LDLR*,* LDLRAP1*, and *PCSK9*, and exon 26 of *APOB* were sequenced in all subjects. Amplicons flanked exons by at least 50 bp from each side to identify splicing mutations. Sequencing was performed using the ABI3100A sequencer at the Molecular Core Facility at the American University of Beirut, following manufacturer's protocol, and as described previously (Nemer et al. 2006; Yehya et al. 2006).

## Results

### Families and subjects

**Figure 1:**
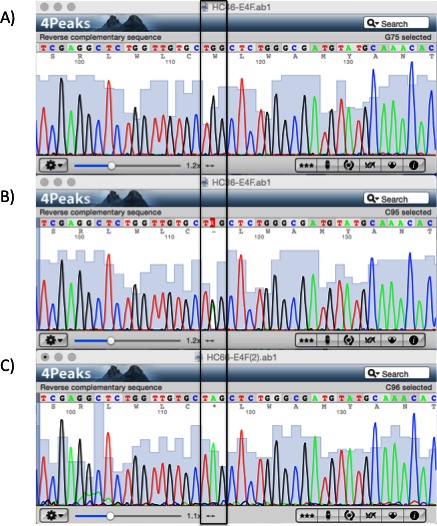
Sequencing results of the LDLRAP1 gene. Chromatograms showing part of exon 4 for the (A) wild‐type, (B) heterozygous, and (C) homozygous p.Q136* variant (boxed).

**Figure 2:**
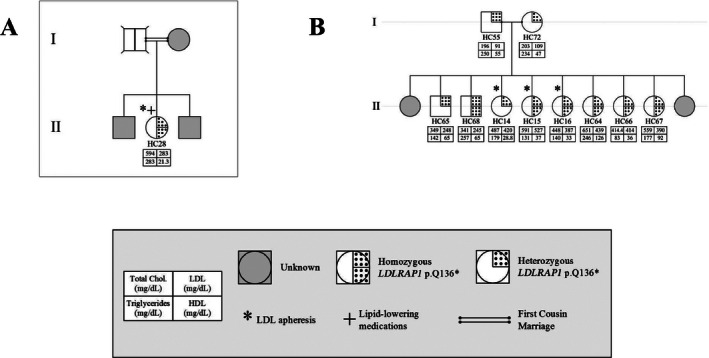
Familial cases of LDLRAP1. Pedigrees of two families A and B with only the p.Q136* variant and the associated phenotypes for each member.

**Figure 3:**
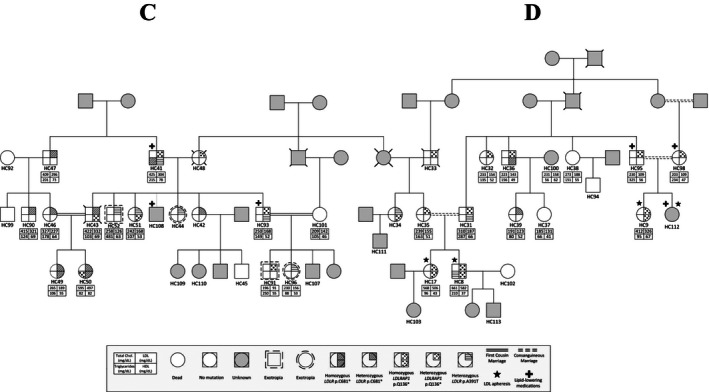
Co‐occurrence of LDLR and LDLRAP1 mutations in familial cases. Pedigrees of two families C and D distantly related with the different genotypes in LDLR (p.C681*, p.A391T) and LDLRAP1 (p.Q136*) and their corresponding phenotypes.

A total of 25 families (a total of 124 individuals) with FH were recruited as part of the Lebanese National FH Database between 2008 and 2012. Twenty‐one families carried mutations in *LDLR* gene and whose phenotype was explained by these mutations. Sanger sequencing of the remaining four families (A, B, C, and D) showed a previously reported mutation in *LDLRAP1* (Figs. 1, 2, 3) and are the focus of this study. Out of 44 subjects recruited from these families, 18 had normal LDL‐C, 9 had mild FH, and 17 had severe FH (Table 1). The median age of subjects was 39.5 years (range 7–70). Their average LDL‐C level was 270.5 mg/dL. Twelve (27%) subjects had xanthomas and 4 (11%) had xanthelasmas.

**Table 1:** Lipid levels and general characteristics of families enrolled in the study

Subjects description	
Families	4
Total subjects	44
With normal LDL	18
With mild FH	9
With severe FH	17
Characteristics	
Age (years)	
Mean	38.4
Median	39.5
Range	7–70
Gender	
Male	16 (36%)
Female	28 (64%)
External manifestations	
Xanthomas	12 (27%)
Xanthelasmas	5 (11%)
Lipid levels	
TC (mg/dL)	
Average	360
Range	185–661
SD	150
LDL (mg/dL)	
Average	270.5
Range	91–582
SD	144.48
HDL (mg/dL)	
Average	56.3
Range	22–126
SD	19.3
TG (mg/dL)	
Average	162
Range	66–325
SD	67

LDL, low‐density lipoprotein; FH, familial hypercholesterolemia; TC, total cholesterol; HDL, high‐density lipoprotein; TG, triglycedride; SD, standard deviation.

Table 1 shows the characteristics of these 44 subjects. The pedigrees are shown in Figures 2 (families A and B) and 3 (families C and D, distantly related).

### Mutations in FH genes in Lebanon

All four families share the same *LDLRAP1* variant, c.C406T that leads to a truncated protein at position 136 (p.Q136*). Eighteen subjects were heterozygous for this variant and only 10 of them had normal LDL‐C levels (Table 2). The average LDL‐C for all heterozygotes was 218 ± 124 mg/dL. Ten patients were homozygous for *LDLRAP1* p.Q136* and all had elevated LDL‐C levels (432 ± 101 mg/dL). In families C and D, mutations in *LDLR* and *LDRAP1* co‐occurred in the same patient in various combinations. Table 3 summarizes the average LDL‐C levels and external manifestations of disease for the different mutation combinations in the two genes.

**Table 2:** List of all different genetic variants causing FH in Lebanon with their respective inheritance pattern

Gene	Variant	Amino acid name	ExAc AF (%)	Number of families	Number of carriers, total (normal LDL‐C)	Heterozygous		Homozygous		Other populations reported
						Number of carriers (with normal LDL‐C)	Average ± SD LDL‐C (mg/dL)	Number of carriers (with normal LDL‐C)	Average ± SD LDL‐C (mg/dL)	
*LDLR*	c.C2043A	p.C681*	8.26E‐06	20	47 (4)	24 (3)	257 ± 111	23 (1)	490 ± 145	Israel, Brazil, UK, USA
*LDLR*	C.G1171A	p.A391T	0.0467	3	9 (4)	9 (4)	197 ± 61	0	NA	South Africa, Russia, Canada, Morocco, Denmark
*LDLR*	c.T1352C	p.I451T	0	1	4 (1)	2 (1)	161 ± 28	2 (0)	596 ± 74	Greece, Netherlands
*LDLR*	C.C2177T	p.T726I	0.006261	1	2 (0	2 (0)	194	0 (0)	NA	USA, UK, Netherlands, Germany, France
*LDLR*	980_981insA	His327fsX5	0	2	3 (0)	0	NA	2 (0)	589 ± 26	Denmark
*LDLRAP1*	c.C406T	p.Q136*	0	4	28 (10)	18 (10)	218 ± 124	10 (0)	432 ± 101	Turkey

FH, familial hypercholesterolemia; LDL‐C, low‐density lipoprotein cholesterol; SD, standard deviation.

**Table 3:** Gentoype/phenotype of all patients with LDLR and/or LDLRAP1 variants

Genotype	*N*	%	LDL (mg/dL) (average ± SD)	XO	XA	LDL apheresis
No mutation	6	13.6	150 ± 22	1/6	0/6	0/6
LDLRAP hetero Q136*	11	25.0	184 ± 116	0/11	0/11	1/11
LDLRAP homo Q136*	9	20.5	391 ± 95	7/9	3/9	5/9
LDLR hetero A391T	3	6.8	156	0/3	0/3	0/3
LDLR hetero C681*	5	11.4	242 ± 88	1/5	1/5	0/5
LDLR homo C681*	1	2.3	189	0/1	0/1	0/1
LDLRAP hetero Q136* + LDLR hetero C681*	2	4.5	238 ± 134	1/2	0/2	0/2
LDLRAP hetero Q136* + LDLR homo C681*	1	2.3	497	1/1	1/1	0/1
LDLRAP hetero Q136* + LDLR hetero A391T	4	9.1	174 ± 11	0/4	0/4	0/4
LDLRAP homo Q136* + LDLR hetero A391T	1	2.3	582	1/1	0/1	1/1
LDLR hetero C681* + LDLR hetero A391T + LDLRAP hetero Q136*	1	2.3	304	0/1	0/1	0/1

*LDLR*, low‐density lipoprotein cholesterol receptor; *LDLRAP1*, LDLR‐associated protein; LDL, low‐density lipoprotein; XO, xanthomas; XA, xanthelasmas; SD, standard deviation.

## Discussion

FH is one of the best described genetic disorders in Lebanon with a high incidence in a small (four millions) and highly consanguineous population. By studying the entire registered FH population in Lebanon, we reported the prevalence of the *LDLR* p.C681* allele variant among affected individuals. In this report we close the genetic gap by unraveling the prevalence of yet another founder mutation in *LDLRAP1* that combined with the *LDLR* variants (p.C681*, p.H327fsX5, p.A391T, and p.I451T) accounts to 100% of the population prevalence of FH in Lebanon.

### Consanguinity, inbreeding, and founder mutations in FH

The high prevalence of consanguineous marriages in the Middle‐East and North African region contributes to a high prevalence of genetic disease, neonate deaths and miscarriages. Both dominant forms due to inbreeding and recessive forms of disease are increased. In Lebanon, the rate of these marriages was estimated to be around 35% based on a cross‐sectional study (Barbour and Salameh 2009). It has been long debated that FH in Lebanon has a founder component linked directly to the high prevalence of consanguineous marriages. The characterization of the Lebanese allele in the *LDLR* gene (p.C681*) in Lebanese expatriates was corroborated recently by our group in showing that it accounts for 80% of the studied families in Lebanon (Table 3). Four percent of the families carried other *LDLR* variants, and the remaining 16% carried the p.Q136* *LDLRAP1* alone or in conjunction with the p.C681*. The high prevalence of consanguinity and inbreeding results in a higher prevalence of homozygous/severe disease as well as in compound heterozygosity of various disease alleles as seen in families C and D.

Five mutations in *LDLR* and one mutation in *LDLRAP1* explain the FH phenotype in 100% of Lebanese patients in the National FH Database (Table 2). All mutations have been reported in other populations, although primarily in patients of Lebanese ancestry. The *LDLR* p.C681* is present in 20 families, either as the only mutation or cosegregating with other FH‐causing mutations, such as in families C and D (Fig. 2).

Founder mutations for FH have been described in many populations, and the most studied one with its relative small population is Sardinia in Italy whereby two *LDLRAP1* variants (p.W22X and Fs144>X170) account for all the cases, and the estimated frequency of the heterozygous carriers is ~1:143 (Filigheddu et al. 2009). The *LDLRAP1* p.Q136* variant was previously described in a Lebanese expatriate family living in Sardinia (Garcia et al. 2001), and in two Turkish families with severely delayed LDL catabolism (Schmidt et al. 1998; Tietge et al. 2003; Soufi et al. 2013). The results of our current study suggest that the variant likely has a founder effect in Lebanon and is the second “Lebanese allele” for FH. It is also possible that the variant migrated between modern day Turkey and Lebanon due to intermarriages during the 600‐year Turkish Empire rule of Lebanon (Ottoman Empire 1299–1922 AC).

### Variable expressivity of FH mutations

Although founder mutations are usually associated with a viable phenotype that could be described as mild allowing the transmission of the genotypes across generations, in most cases of FH whether dominant or recessive, the clinical features appear very early on in the patients as is the case of the Lebanese *LDLR* allele (Khachadurian 1964; Fahed et al. 2011). Management of such cases could thus be ameliorated based on family history. The big challenge remains, however, the variable expressivity of the phenotype despite sharing the same genotype.

The gaps in establishing phenotype–genotype correlations might be overcome by whole exome sequencing of all affected and nonaffected familial cases. Although no single variant modifier allele has been linked so far to amelioration or exacerbation of the clinical phenotype when concomitant with a FH genotype, some variants have been previously reported in carriers of the dominant hypercholesterolemia mutations with low levels of LDL‐C (Brusgaard et al. 2010; Huijgen et al. 2012). These variants affect mainly *PCSK9*,* APOB*, and *ANGPTL3* (data not shown); total loss of the latter causes familial hypolipidemia (Musunuru et al. 2010). In our cohort of patients we failed to detect any potential causative variant in the three genes except for the *PCSK9* variant (p.R46L) which was previously associated with low LDL‐C levels in normal and hypercholesterolemia patients (Humphries et al. 2009), and was found in only one patient (HC28 in Fig. 2A). This patient has a very severe phenotype and thus the p.R46L variant has no effect on a case where the adaptor protein is presumably not produced, confirming thus, the hypothesis that the action of PCSK9 is dependent on its interaction directly or indirectly via the *LDLR* to *LDLRAP1* (Soutar and Naoumova 2007; Fahed and Nemer 2011; Urban et al. 2013). This contrasts the results obtained for the p.L21dup *PCSK9* allele found in patients with the p.C681* variant and conferring low LDL‐C levels (Abifadel et al. 2009).

### Failure of the dominant and recessive dichotomy in FH

The concomitant presence of the two forms of FH, recessive and dominant, within the same family represents a major challenge for treatment and follow‐up because of the complexity of the phenotype. Only two cases such as the ones we are hereby reporting are published. The first reported a double heterozygous case for both *LDLR* and *LDLRAP1* suggesting that the additional *LDLRAP1* variant confers more severity to the phenotype observed in the *LDLR* variant alone (Tada et al. 2011). The second involves a Turkish family with the same homozygous p.Q136* *LDLRAP1* variant as in this report, in combination with the p.Q254P *LDLR* variant (Soufi et al. 2013). The authors noted that the phenotype observed in the patients with combined heterozygous *LDLR* variant and *LDLRAP1* homozygous variant is more severe than the one observed in the homozygous *LDLR* variant alone. In our case, we could not identify a universal pattern but we noticed that the combination of homozygous variant of one gene with the heterozygous variant of the other gene results in a drastic increase of LDL‐C levels as is the case of patient HC50 in family C and patient HC8 in family D (Fig. 3). Nevertheless, the presence of three heterozygous variants does not equally increase the LDL‐C levels as in the case of HC41 (Fig. 3).

**Figure 4:**
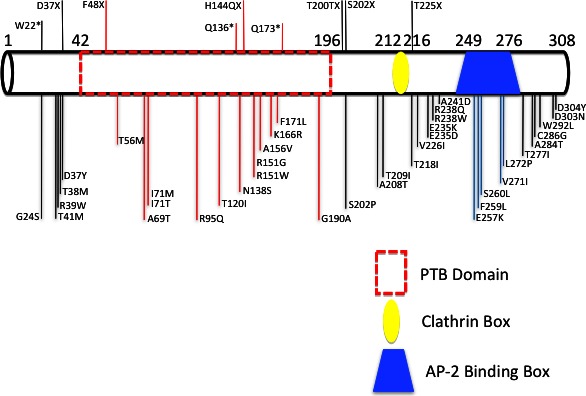
Diagram of all LDLRAP1 mutations ever reported and their location on the protein.

While the gene *LDLRAP1* is presumed to act in a recessive fashion, we report heterozygous carriers of p.Q136* with a severe FH phenotype equivalent to the homozygous carriers (Figs. 2, 3). This goes in parallel with our previous findings on the heterogeneous phenotypes observed in patients with the *LDLR* p.C681* variant (Fahed et al. 2012). While gene dosage of *LDLR* and *LDRAP1* is playing a role in dictating the serum LDL‐C levels, it is not possible to obtain enough statistical power with current numbers to better understand these associations. Other players such as environmental, genetic modifiers, and treatment are also likely confounding the relationship with LDL‐C levels. Finally, the polygenic nature of the disease is to be taken into consideration whereby in some cohorts up to 60% of the cases are still with no genetic clues (Talmud et al. 2013; Futema et al. 2015) (Fig. 4).

Overall, a better understanding of the different genetic variants in the physiological context is required to better define the role of each of the players in cholesterol synthesis and uptake process in a particular patient. Whole‐genome sequencing of large cohorts of FH are needed to establish statistical power for better understanding of LDL‐C level modifiers. Meanwhile, cascade genetic screening allows identification of cases early and serum LDL‐C level remain the best predictor of cardiovascular disease risk (Fahed et al. 2014).

## Conflict of Interest

None declared.
